# Supplementary material for: Splice-Junction-Based Mapping of Alternative Isoforms in the Human Proteome
Source: Cell Rep. Author manuscript; Available in PMC 2020 Jan 15. (PMC6961840; doi:10.1016/j.celrep.2019.11.026)
Supplement: 2 [file NIHMS1546469-supplement-2.zip › DF1/PXD010154/Liver/Liver_51_SCARF1_ALQTQAAVSPASR.pdf]

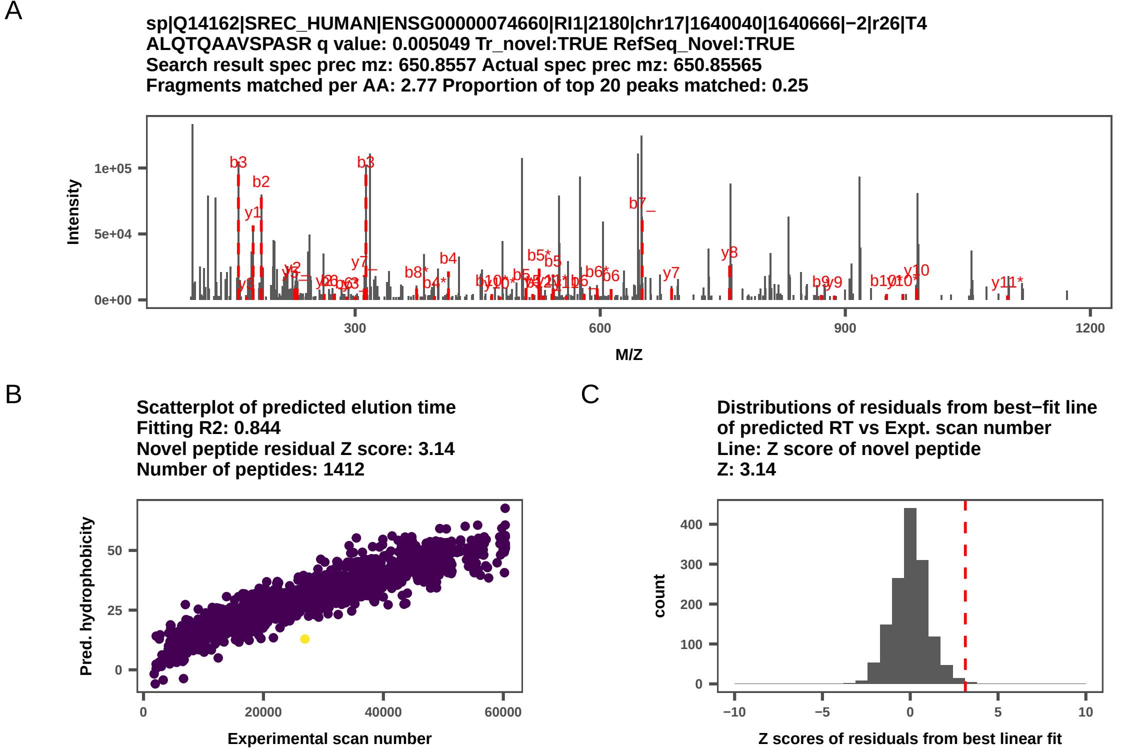
A
sp|Q14162|SREC_HUMAN|ENSG00000074660|RI1|2180|chr17|1640040|1640666|−2|r26|T4
ALQTQAAVSPASR q value: 0.005049 Tr_novel:TRUE RefSeq_Novel:TRUE
Search result spec prec mz: 650.8557 Actual spec prec mz: 650.85565
Fragments matched per AA: 2.77 Proportion of top 20 peaks matched: 0.25
Intensity
1e+05
5e+04
0e+00
300
600
900
1200
M/Z
b3
b2
y1
b3
b7_
y8
b4
b8*
b4*
y7
y10
y11*
B
Scatterplot of predicted elution time
Fitting R2: 0.844
Novel peptide residual Z score: 3.14
Number of peptides: 1412
Pred. hydrophobicity
50
25
0
0
20000
40000
60000
Experimental scan number
C
Distributions of residuals from best−fit line
of predicted RT vs Expt. scan number
Line: Z score of novel peptide
Z: 3.14
count
400
300
200
100
0
−10
−5
0
5
10
Z scores of residuals from best linear fit
